# Hadronic Higgs production through NLO $+$ PS in the SM, the 2HDM and the MSSM

**DOI:** 10.1140/epjc/s10052-015-3462-1

**Published:** 2015-06-10

**Authors:** Hendrik Mantler, Marius Wiesemann

**Affiliations:** TH Division, Physics Department, CERN, 1211 Geneva 23, Switzerland; Physik-Institut, Universität Zürich, 8057 Zurich, Switzerland

## Abstract

The next-to-leading order (NLO) cross section of the gluon fusion process is matched to parton showers in the MC@NLO approach. We work in the framework of MadGraph5_aMC@NLO and document the inclusion of the full quark-mass dependence in the Standard Model (SM) as well as the state-of-the-art squark and gluino effects within the Minimal Supersymmetric SM  embodied in the program SusHi. The combination of the two programs is realized by a script which is publicly available and whose usage is detailed. We discuss the input cards and the relevant parameter switches. One of our focuses is on the shower scale which is specifically important for gluon-induced Higgs production, particularly in models with enhanced Higgs-bottom Yukawa coupling.

## Introduction

Higgs production proceeds predominantly through gluon fusion in a large number of theories, including the Standard Model (SM). The recently discovered resonance [1, 2] in searches for a Higgs boson is fully consistent with the SM picture,^1^ so far. Still, the measured Higgs boson may be embedded in an enlarged Higgs sector with respect to the one of the SM which predicts only a single physical particle breaking the electro-weak symmetry. Two-Higgs doublet models (2HDM’s) such as the Minimal Supersymmetric SM (MSSM) are among the most popular theories with enlarged Higgs sectors. Such theories inevitably require the existence of further physical Higgs particles. A 2HDM predicts three neutral Higgs bosons: a light (*h*) and a heavy (*H*) scalar, and a pseudo-scalar (*A*); as well as two charged Higgs particles ($H^\pm$). Almost all 2HDM and MSSM scenarios that are in agreement with the experimental bounds feature a light scalar which is SM-like in its couplings to vector bosons and fermions, while the other Higgs bosons are heavier and, therefore, escaped detection up to now. Indeed, the experimental search for other Higgs resonances is one of the major focuses regarding the discovery of physics beyond the SM (BSM) in the second run of the Large Hadron Collider (LHC).

Higgs production through gluon fusion is mediated by a colored particle. In the SM, the top quark gives the dominant contribution to the cross section [6–8]. While also the bottom quark gives a sizable contribution, the effects due to other quarks are small and therefore usually neglected. In the 2HDM and the MSSM the Higgs-bottom Yukawa coupling can be enhanced with respect to the one of the top quark and the bottom loop may even constitute the dominant contribution to the cross section. In those models it is stringently required to include the bottom-quark contribution.^2^ The gluon fusion cross section is known at the next-to-leading order (NLO) in the SM including top- and bottom-mass effects [18, 19], in the 2HDM and in the MSSM including contributions from squarks and gluinos [20–34]. For the top quark, an effective field theory approach can be applied in which the top quark is considered to be infinitely heavy and can be integrated out from the full theory. In this approximation, Higgs production has been calculated up to the next-to-NLO (NNLO) inclusively [35–37] as well as fully differential [38–40]. Electro-weak contributions and effects beyond NNLO in the heavy-top approximation have been studied in Refs. [41–50] for example, while there was a large effort [51, 52] to push the accuracy to next-to-NNLO ( N$^3$LO) which has been succeeded very recently [53]. Finite top-mass effects have been shown to be small for both the inclusive cross section at the NNLO [54–59] and differential quantities [60, 61] as long as no kinematical scale (such as the transverse momentum of a particle) that is not integrated out exceeds the top-mass threshold.

The full dependence of the top- and the bottom-mass at the NLO has been included so far in a POWHEG-type [62] matching to parton showers (PSs) [33], the analytically resummed transverse momentum spectrum of the Higgs boson at NLO $+$ NLL [63], a MC@NLO-type [64] matching to the Herwig Monte Carlos [65–67], the NNLO $+$ NNLL jet-vetoed [68] and the fully differential NNLO [69] cross section; and in some approximated form recently also in the NNLOPS approach [70, 71]. Furthermore, the 2HDM as well as supersymmetric effects from squarks and gluinos within the MSSM [20–34] have been implemented in the first two approaches from that list [33, 72]. In this manuscript, we report on a new implementation of NLO QCD corrections in the SM, 2HDM and MSSM applying the MC@NLO-type matching to both Herwig and Pythia showers. We work in the framework of MadGraph5_aMC@NLO [73] and combine its capabilities with the corresponding amplitudes provided by SusHi [74]. The linking of SusHi to MadGraph5_aMC@NLO is realized by a script^3^aMCSusHi. Its usage as well as the application of the combined code to obtain cross section predictions in the SM, the 2HDM and the MSSM is detailed in this paper.^4^

The manuscript is organized as follows: In Sect. 2 we present a brief overview of the elements of the computation at hand. Section 3 is dedicated to introduce aMCSusHi and is separated in three parts which cover: how to use the script (Sect. 3.1), how to run the resulting code (Sect. 3.2) and how to treat the shower scale (Sect. 3.3). We will show a brief application of the code to phenomenological results in Sect. 4 and conclude in Sect. 5.

## Outline of the calculation

The goal of this paper is to present a tool which allows for the computation of arbitrary infra-red safe differential observables at both the parton and the hadron level for the production of neutral Higgs bosons via gluon fusion in the SM, the 2HDM and the MSSM by matching the NLO cross section to a shower.

**Fig. 1:**
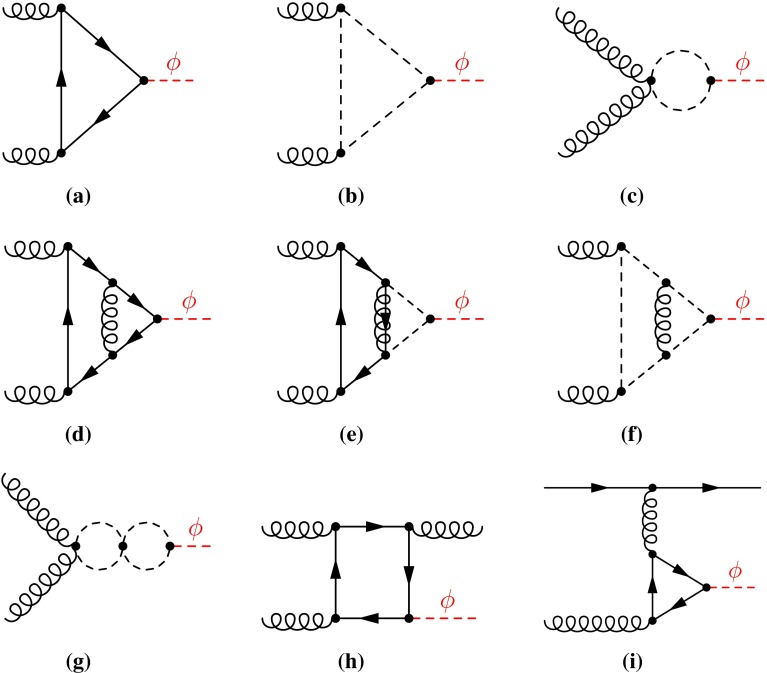
A sample of Feynman diagrams for $gg\rightarrow \phi$ contributing to the NLO cross section; **a**–**c** LO, **d**–**g** virtual, and **h**, **i** real corrections. The graphical notation for the *lines* is: *solid straight* $\widehat{=}$ quark; *spiraled* $\widehat{=}$ gluon; *dashed* $\widehat{=}$ scalar (squark or Higgs); *spiraled* with *line* $\widehat{=}$ gluino

The relevant NLO matrix elements are taken from Ref. [74], which include both SM-like contributions and sbottom, stop and gluino effects. Examples of corresponding Feynman diagrams are illustrated in Fig. 1. They are combined and matched to a parton shower by the well-known MC@NLO-method. The matched cross section in the MC@NLO framework can be written symbolically as

1 $$\begin{aligned}&\left( \frac{\mathrm{d}\sigma }{\mathrm{d}O}\right) _{\text {MC@NLO}}=\int \mathrm{d}\Phi _n\left[ B_n+V_n+\int \mathrm{d}\Phi _{1}^{\tiny \text{ MC }}\,K^{\tiny \text{ MC }}_{n+1}\right] \mathcal I^{\tiny \text{ MC }}_n(O)\nonumber \\&\quad \quad +\int \left[ \mathrm{d}\Phi _{n+1}R_{n+1}-\mathrm{d}\Phi _{n+1}^{{\tiny \text{ MC }}} K^{\tiny \text{ MC }}_{n+1}\right] \mathcal I^{\tiny \text{ MC }}_{n+1}(O)\,, \end{aligned}$$

where $B_n$ determines the Born-level cross section, $V_n$ the virtual (including mass factorization) and $R_{n+1}$ the real corrections; $K^{\tiny \text{ MC }}_{n+1}$ is the Monte Carlo subtraction term, with the same IR poles as $R_{n+1}$, the Monte Carlo phase space $\mathrm{d}\Phi _{n+1}^{\tiny \text{ MC }}$ tends to $\mathrm{d}\Phi _{n+1}$ in the IR limits, and $\mathrm{d}\Phi _{1}^{\tiny \text{ MC }}=\mathrm{d}\Phi _{n+1}^{\tiny \text{ MC }}/\mathrm{d}\Phi _n$. The quantity $\mathcal I_n^{\tiny \text{ MC }}(O)$ is the shower spectrum for observable *O*, as obtained by running the shower starting from an *n*-body configuration.

The cross section at Born level is derived from the LO diagrams for $gg\rightarrow \phi$ where $\phi \in \{h,H,A\}$; see e.g. Fig. 1a–c. The NLO virtual and real corrections are governed by diagrams like the ones shown in Fig. 1d–g and h, i, respectively, and similar ones with quark loops replaced by squark loops.

Equation (1) is implemented for all standard parton showers [76–81] in the fully automated framework MadGraph5_aMC@NLO. This code determines NLO QCD  corrections to arbitrary scattering processes at the LHC. On the basis of UFO models [82], the code even allows one to carry out computations in any theory beyond the SM in a general manner as soon as the renormalization is known and implemented in a UFO model; see Refs. [73, 83–85] for further information. However, the Higgs production mode through gluon fusion is special, being loop-induced already at the LO. Such processes cannot be handled in a fully automated manner by any code to date, since it requires the automation of two-loop amplitudes which is beyond current technology. Therefore, we have treated Higgs production through gluon fusion in the SM, 2HDM and MSSM as a special case, by linking the relevant amplitudes from SusHi. Furthermore, as far as the MSSM is concerned SusHi requires a link to FeynHiggs [86–96] which evaluates the corresponding Higgs masses and couplings in user defined scenarios. Setting up the SusHi amplitudes in MadGraph5_aMC@NLO is handled by a publicly available script called aMCSusHi, which is automated to create the $gg\rightarrow \phi$ process folder; download SusHi and FeynHiggs; compile, install and link them; and replace the relevant amplitudes in the process folder. In the upcoming section, we describe the application of the script and explain the necessary steps to obtain phenomenological results.

## aMCSusHi script

This code is based on MadGraph5_aMC@NLO and SusHi. For further information on theses codes we refer the reader to corresponding publications [73, 74]. After using the script to set up the code, we will focus on the relevant user inputs to obtain phenomenological predictions for Higgs cross sections in the SM, the 2HDM and the MSSM.

### Usage of the script

The aMCSusHi script is available for download from the website https://cp3.irmp.ucl.ac.be/projects/madgraph/wiki/aMCSushi. It is fully automatic in setting up the $gg\rightarrow \phi$ process folder which includes downloading, installing and linking FeynHiggs and SusHi. At first, a dummy HEFT process folder for $gg\rightarrow h$ in the five-flavor scheme at NLO (without the virtuals) is created;^5^ then the HEFT amplitudes in MadGraph5_aMC@NLO are consistently replaced by the ones from SusHi (including the virtuals). The setup requires only a single call of the aMCSusHi script:

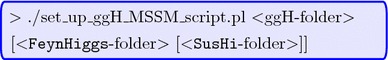



The first argument is mandatory and determines the path to the process folder for $gg\rightarrow \phi$ that is generated by the script. The only requirement is that this folder has to be defined as a sub-folder of the MadGraph5_aMC@NLO directory. The second and third arguments are optional. If there are compiled versions of FeynHiggs and SusHi available on your computer you can give the names of the folders that contain the files libFH.a and libsushi.a, respectively. When executed with two (one) argument(s) the script will ask whether it should automatically download and install SusHi (and FeynHiggs). The script will always download the latest versions of these codes. While running, the script requires some user inputs: It asks whether or not SusHi (and FeynHiggs) should be downloaded, in which folder they should be installed (default is inside the $\langle$ggH-folder$\rangle$) or where to find SusHi (and FeynHiggs) if already installed. The user is simply required to follow these on-screen instructions. Furthermore, the script creates log-files in the working directory for the download (“XX_curl.log”), the configure command (“XX_conf.log”) and the compilation (“XX_make.log”), where XX $=$ FH for FeynHiggs and XX $=$ SusHi for SusHi. These files are supposed to give complementary information for any kind of troubleshooting.^6^ Further information about the aMCSusHi script provides the README file.

### Running the code

Once the $gg\rightarrow \phi$ process folder has been set up by the script, the run can be started directly from the $\langle$ggH-folder$\rangle$ by typing

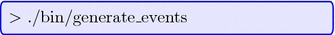



and following the usual steps in MadGraph5_aMC@NLO to choose the run-modes (order, shower or fixed-order, madspin). Before running the code, one may want to modify the input settings. In the following we will discuss the differences between aMCSusHi and the ordinary MadGraph5_aMC@NLO code regarding the input files.

The input cards can be found under $\langle$ggH-folder$\rangle$/Cards/, where the param_card.dat, run_card.dat and shower_card.dat contain all the essential information. The run card controls the usual parameters, e.g. the renormalization ($\mu _\mathrm{R}$) and factorization scale ($\mu _\mathrm{F}$). Note that by default the flags fixed_ren_scale and fixed_fac_scale are set to false, so that these two scales are chosen on an event-wise basis. Their values are specified in the last routine of $\langle$ggH-folder$\rangle$/SubProcesses/setscales.f which, due to the default option $\mathtt{dynamical\_scale\_choice}=-1$ in the run card, sets $\mu _\mathrm{R}=\mu _\mathrm{F}=H_T/2\equiv 1/2\,\sum _{i}(m_i^2+p_T^2(i))^{1/2}$, where *i* runs over all final state particles and $m_i$ and $p_T(i)$ are their mass and transverse momentum, respectively. This choice is reasonable, since it respects effects from hard radiation and corresponds to a value of $m_{\phi }/2$ in the soft/collinear limit which is the current recommendation for the total inclusive $gg\rightarrow \phi$ cross section [3, 5].

Also the shower card in aMCSusHi contains no new information and has the usual functionality. Since MadGraph5_aMC@NLO supports all standard parton showers, for the first time Pythia6 and Pythia8 can be applied at NLO $+$ PS to SM Higgs production in the full theory in the MC@NLO framework. In general, it is advisable to apply the most recent versions of the showers for meaningful physics runs.

The param_card.dat, on the other hand, receives some significant changes by the aMCSusHi script. The new version basically combines the orignial parameter card from MadGraph5_aMC@NLO with the input file from SusHi which are both written in the SUSY Les Houches accord (SLHA) format [97] and, therefore, easily connectable. We will address the different options in the param_card.dat in more detail, since there are a number of changes and some of the original parameters lose their functionality. In the SLHA format inputs are organized in blocks which have different entries that are characterized by a number. For simplicity, we introduce the following short-hand notation: Block example[i] corresponds to entry *i* in Block example. E.g., entry 25 of Block mass (Block mass[25]) in the SLHA format is devoted to the Higgs mass in the SM, which is required as an input in the param_card.dat. A typical parameter card of aMCSusHi in the SM is shown below:

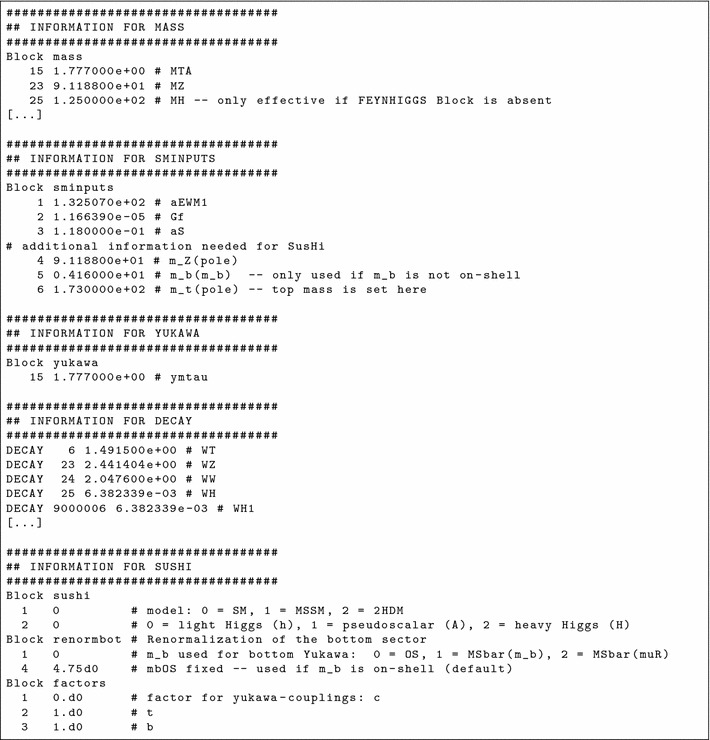



Most of the inputs are self-explanatory due to the comments initiated by the hash symbol # after the entries. Furthermore, the standard SLHA blocks match the universal convention of Ref. [97]. Some of the inputs, though, require further comments. In the Block mass all parameters have the expected function, except for the top and the bottom mass, Block mass[6] and Block mass[5], respectively. While the former only affects and is required for the shower, the latter can be omitted completely. Instead, due to the link to SusHi the top mass that is used for the top loop and the top Yukawa is set in Block sminputs[6] and is expected to be on-shell. For the bottom mass, on the other hand, SusHi allows for three different choices: on-shell scheme or $\overline{\text {MS}}$ scheme with $m_b(m_b)$ or $m_b(\mu _\mathrm{R})$, which can be switched in Block renormbot[1] by a value between 1 and 3.^7^ Also here the recommendation is to use the on-shell scheme which according to Refs. [18, 98] ensures the cancelation of large logarithms $\ln (m_{h}/m_b)$ at NLO QCD, while the $\overline{\text {MS}}$ scheme does not, due to an incomplete resummation of these terms. The on-shell $m_b$ value is determined by Block renormbot[4], while when the $\overline{\text {MS}}$ scheme is chosen the corresponding input of $m_b(m_b)$ is set in Block sminputs[5]. The other entries of Block sminputs again have the same impact as in the usual MadGraph5_aMC@NLO code. The same is true for Block yukawa. For the decay of light and heavy Higgs bosons one may specify a finite width of the Higgs boson in the respective BSM scenario by using Decay 25 irrespective of whether the light or the heavy Higgs boson is considered. At this point we shall remark that the particle identification number in the generated event files is always 25 regardless of the Higgs boson under consideration.^8^ This is irrelevant for the production (which is correctly computed through the SusHi amplitudes), but it plays a role for the decay where the shower will consider particle 25 to be the light Higgs, which is indeed fine for any scalar Higgs, but a problem for pseudo-scalar ones. Therefore, decays of a pseudo-scalar Higgs are currently not supported in the official version of aMCSusHi. A user interested in decaying the pseudo-scalar Higgs is strongly encouraged to contact us.

The other parameters are relevant to SusHi. Block sushi[1] chooses the model with the three options SM (Block sushi[1] $=0$), MSSM (Block sushi[1]$=1$) and 2HDM (Block sushi[1] $=2$). The second entry of Block sushi determines the Higgs boson: light Higgs (Block sushi[2] $=11$ or 0), pseudo-scalar Higgs (Block sushi[2] $=21$ or 1) and heavy Higgs (Block sushi[2] $=12$ or 2). The choice of the masses of the relevant Higgs bosons depends on the model. As stated before, Block mass[25] sets the Higgs mass in the SM. In the 2HDM, this entry corresponds to the mass of the light Higgs boson, while Block mass[35] and Block mass[36] specify the input for the heavy and the pseudo-scalar Higgs, respectively. All other 2HDM inputs are set in the information for SusHi. For reference, we give an example of the corresponding inputs for a heavy Higgs in the 2HDM below^9^:

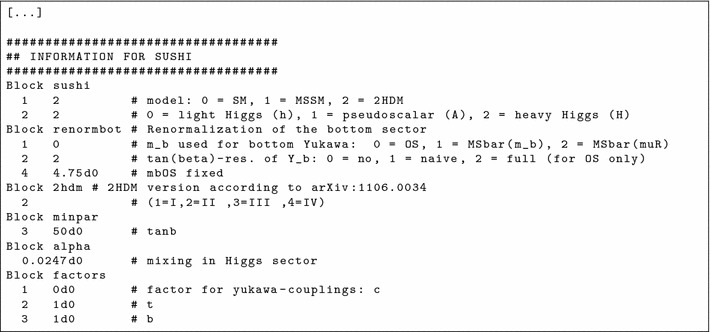



Additionally to the inputs which we defined already for the SM the following parameters have to be set in the 2HDM: Block renormbot[2] specifies whether or not a resummation^10^ of terms enhanced by $\tan (\beta )$ is applied through reweighting of the bottom Yukawa coupling as described in Ref. [74]; Block 2hdm determines which type of the 2HDM is used; the value of $\tan (\beta )$ is set through Block minpar[3]; and the mixing angle $\alpha$ corresponds to the entry in Block alpha.

The computation of MSSM Higgs cross sections requires Block extpar, Block feynhiggs and Block renormsbot in addition, which fix the parameters of the third family of quarks and squarks, determine the FeynHiggs inputs and yield information on the renormalization of the sbottom section, respectively. We will not provide any further information on these blocks, instead, we refer to the SusHi manual [106] and the FeynHiggs man pages [107]. Moreover, Block alpha can be omitted in the MSSM and the Higgs masses in Block mass have no effect, since they are determined by FeynHiggs, once Block feynhiggs is present. The MSSM Higgs mass that has been computed and applied in the run is provided to the user in Block mass[25] of the parameter card, which will be overwritten by the mass of the respective Higgs boson at the beginning of each MSSM run.

So far we did not comment on the Block factors. It allows one to turn on and off individual contributions in all models. In fact, it even provides the possibility to rescale the respective Yukawa couplings by choosing values different from 0 and 1. With Block factors[1] one can include the charm quark in the computation. This requires one to specify its $\overline{\text {MS}}$ mass $m_c(m_c)$ in Block sminputs[8] which is then translated to its on-shell mass. Furthermore, Block factors[2] and Block factors[3] multiply the top and the bottom Yukawa, respectively. In the MSSM, the stop Yukawa is rescaled by Block factors[4] and the sbottom one by Block factors[5].

For further information on the input cards we refer the reader to Ref. [73] of MadGraph5_aMC@NLO, the manual of SusHi [106] and the man pages of FeynHiggs [107]. Three example parameter cards are provided in the folder $\langle$ggH-folder$\rangle$/Cards; one for the SM (param_card.dat_SM), the 2HDM (param_card.dat_2HDM_scenB) and the MSSM (param_card.dat_MSSM_mhmodp). They match the scenarios that we study in the result section of this paper.

### Choosing different shower scales

The choice of the shower scale is a very peculiar one in the gluon fusion process. In presence of the bottom-quark loop, factorization of soft and collinear radiation maybe spoiled at scales significantly smaller than the Higgs boson mass. This was pointed out by Ref. [69] in the context of analytic transverse momentum resummation. On the other hand, these terms might well be treated as a finite remainder as long as their impact remains moderate [68].

Due to their additive matching of the resummed low-$p_T$ region with the fixed-order distribution valid at large transverse momenta, analytic $p_T$-resummation and the MC@NLO-method are quite similar. In both cases there is a scale associated with that matching, the resummation scale $Q_{\text {res}}$ and the shower scale $Q_{\text {sh}}$, respectively. These scales can be interpreted as transition scales that separate the soft/collinear from the hard physics, very similar to the factorization scale of the PDFs. In other words, they define the range where resummation, and therefore the shower in MC@NLO, takes effect. Their value has to be chosen of the order of the typical scale of the problem.

In gluon fusion, the typical scale depends on the quark considered in the loop. Since $m_t\sim m_{\phi }$, there exist only two relevant scales for the top-quark loop ($m_{\phi }$ and $p_T$) and the shower scale can be chosen of the order of the Higgs mass. When considering the bottom loop, on the other hand, we face a three-scale problem ($m_{\phi }$, $m_b$, and $p_T$) which has not been solved to date. However, it has been suggested [69] to apply a lower transition scale to the bottom contribution, which, in particular, respects the fact that soft/collinear factorization is valid only up to smaller scales for the bottom loop. In Ref. [72] it was further proposed to separate three contributions according to their Yukawa couplings: the square of the top and the bottom, and their interference; and choose separate shower/resummation scales for all of them. This splitting allows for a model independent treatment of the problem by a rescaling of the individual contributions with the respective top and bottom Yukawas of a specific scenario in the 2HDM as well as the MSSM when neglecting squark effects. In the literature, two pragmatic approaches with physical motivation have been presented [72, 108] to determine separate scales for the three contributions. Their comparison will be studied elsewhere [109]. When studying phenomenological results in Sect. 4 we will apply the scales from Ref. [72] (referred to as “HMW” in what follows).

The separation of the bottom contribution (including the interference) from the top one with different shower scales ($Q_b$ and $Q_t$, respectively) requires three runs in MadGraph5_aMC@NLO, which have to be combined as follows:

2 $$\begin{aligned} \sigma (Q_t,Q_b)=\sigma _t(Q_t)+\sigma _{t+b}(Q_b)-\sigma _t(Q_b). \end{aligned}$$

To obtain all three contributions of different Yukawa origin with different scales that allows for a model independent treatment, on the other hand, MadGraph5_aMC@NLO has to be run five times:

3 $$\begin{aligned} \sigma (Q_t,Q_b,Q_{tb})= & {} \sigma _t(Q_t)+\sigma _b(Q_b)+\sigma _{t+b}(Q_{tb})\nonumber \\&-\sigma _t(Q_{tb})-\sigma _b(Q_{tb}). \end{aligned}$$

The scales $Q_t$, $Q_b$, and $Q_{tb}$ determine the scale for top, the bottom and their interference, respectively. As indicated before, the individual contributions can be separated using the Block factors in the parameter card.

The shower scale in MadGraph5_aMC@NLO cannot simply be accessed through the input cards, since it requires an advanced user to be familiar with its specific treatment in the code. MadGraph5_aMC@NLO does not use a simple fixed scale for $Q_{\text {sh}}$, instead, it statistically extracts the shower scale from a distribution peaked at a specific value. The user can only change the range of the interval of the distribution which of course also affects the peak. Therefore, we identify $Q_t$, $Q_b$, and $Q_{tb}$ in Eqs. (2) and (3) with the peak of the respective shower scale distributions.

The so-called shape parameters define the interval of the distribution from which the shower scale is picked on an event-wise basis. They can be specified in the include file $\langle$ggH-folder$\rangle$/SubProcesses/madfks_mcatnlo.inc, where the relevant part is given by (MadGraph5_aMC@NLO default values):

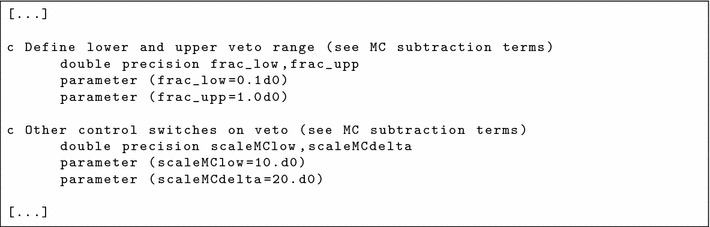



The parameters frac_low and frac_upp are used to compute the upper and lower bounds of the $Q_{\text {sh}}$ distribution, which will be explained in more detail below, while scaleMClow allows one to set an absolute value of the lower bound on $Q_{\text {sh}}$ and scaleMCdelta is used to apply a minimal value to the size of the distribution interval. In formulas the interval is defined by^11^

4 $$\begin{aligned} Q_{\text {min}}\le & {} Q_{\text {sh}}\le Q_{\text {max}}, \quad \text {with} \nonumber \\ Q_{\text {min}}= & {} \max (\mathtt{frac\_low}\cdot \sqrt{s_0},\mathtt{scaleMClow}) \quad \text {and }\\ Q_{\text {max}}= & {} \max (\mathtt{frac\_upp}\cdot \sqrt{s_0},Q_{\text {min}}{+}\mathtt{scaleMCdelta}),\nonumber \end{aligned}$$

where $s_0$ is the Born-level partonic center of mass energy squared. Evidently, scaleMClow and scaleMCdelta only take effect if the interval obtained through frac_low and frac_upp does not meet the corresponding restrictions. The corresponding $Q_{\text {sh}}$ distribution is peaked around

5 $$\begin{aligned} Q_{\text {peak}}\sim \frac{(\mathtt{frac\_low}+\mathtt{frac\_upp})}{2}\,\sqrt{\langle s_0\rangle }. \end{aligned}$$

For a $2\rightarrow 1$ process like gluon fusion this relation is an identity and $\sqrt{s_0}$ equals the mass of the final state particle, i. e., the Higgs mass in the case of gluon fusion. To change the peak-value to its half, e. g., for shower scale variations, one can simply divide frac_low and frac_upp by a factor of two. In this sense, it is convenient to keep the ratio between frac_low and frac_upp a constant, which in the default setup of MadGraph5_aMC@NLO is a factor of ten. Under this prerequisite, in order to choose a specific shower scale $Q_{\text {sh}}$ for $gg\rightarrow \phi$ we simply have to determine

6 $$\begin{aligned} \mathtt{frac\_upp}= \frac{2}{1.1}\cdot \frac{Q_{\text {sh}}}{m_{\phi }} \quad \text {and}\quad \mathtt{frac\_low}=\frac{\mathtt{frac\_upp}}{10}.\nonumber \\ \end{aligned}$$

Here and in what follows, we associate $Q_{\text {peak}}$ with the shower scale $Q_{\text {sh}}$ and vice versa unless stated otherwise. After modifying the corresponding include file accordingly, MadGraph5_aMC@NLO has to be recompiled. This can be achieved by typing

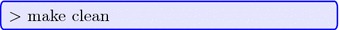



inside the $\langle$ggH-folder$\rangle$, which forces a recompilation during the next run of the code.

In Sect. 4 we show some applications of the aMCSusHi code and study the effect of different treatments of the shower scales.

## Results: brief application

The $gg\rightarrow \phi$ process folder created by the aMCSusHi script preserves all the highly convenient features that come with MadGraph5_aMC@NLO. Besides many others, this entails an interface to the most common showers, the fully automatic determination of scale and PDF variations without any extra-costs of computing time [110], the creation of any number of observables with a single run and analysis routines available for the most important processes including the gluon fusion Higgs production mode.

**Fig. 2:**
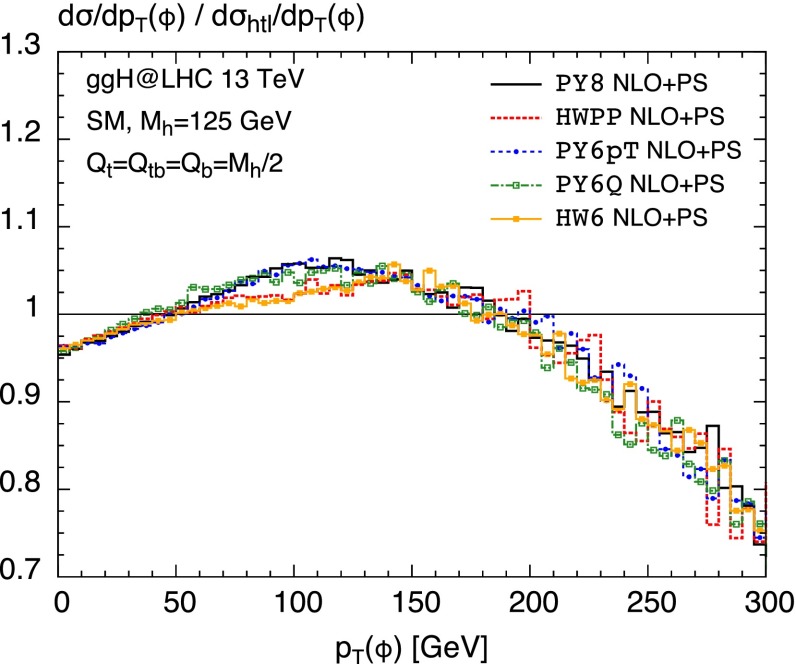
Transverse momentum distribution of a SM Higgs at NLO $+$ PS in the full theory normalized to the one in the heavy *top* effective field theory for different Monte Carlos: Pythia8 (*black solid*), Herwig++  (*red*, *dotted*), Pythia6 $p_T$-ordered (*blue*, *dashed* with *points*), Pythia6 *Q*-ordered (*green*, *dash-dotted* with *open boxes*) and Herwig6 (*yellow*, *solid* with *filled boxes*)

aMCSusHi allows one to compute gluon-induced Higgs production including the complete dependence on the quark masses in the SM for the first time in a MC@NLO-type matching applying all versions of Pythia and Herwig Monte Carlos. While previous computations did only feature the Herwig showers [65–67], phenomenological results exist to our knowledge only for Herwig6 [111]. As a first application we therefore study the impact of different showers on the top- and bottom-mass effects with respect to the heavy-top approximation at the 13 TeV LHC. For this purpose, Fig. 2 shows the ratio of the NLO $+$ PS computation including mass effects and the corresponding cross section in the heavy-top limit as a function of the transverse momentum of the Higgs for different Monte Carlos: Pythia8 (black, solid), Herwig++  (red, dotted), Pythia6 $p_T$-ordered (blue, dashed with points), Pythia6 *Q*-ordered (green, dash-dotted with open boxes) and Herwig6 (yellow, solid with filled boxes). We apply the MSTW2008 $68$ % CL NLO PDF set [112] with the corresponding value of the strong coupling constant. The shower scale has been chosen as $Q_{\text {sh}}=m_{h}/2$ in all cases, while for $\mu _\mathrm{F}$ and $\mu _\mathrm{R}$ we use the defaults specified in Sect. 3.2. Clearly, the mass effects are hardly dependent on the specific Monte Carlo, which is particularly evident at small ($p_T\lesssim 50$ GeV) and large ($p_T\gtrsim 150$ GeV) transverse momenta. Nevertheless, there are some visible differences in the intermediate region which consistently discriminate the Herwig from the Pythia showers. Overall, they are at most $5\,\%$ though and therefore still moderate.

**Fig. 3:**
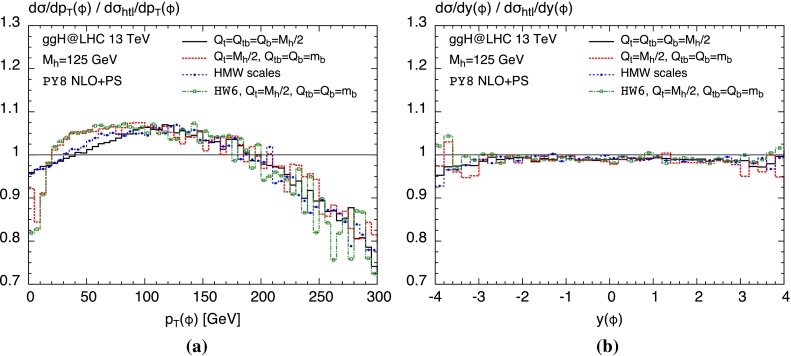
**a** Same as Fig. 2, but for different choices of the shower scales; see text for details. **b** Corresponding *plot* for the rapidity distribution of the Higgs

Figure 3 shows the effects of quark masses with respect to the heavy-top approximation as well, but for different choices of the associated shower scales. In all cases, the denominator and therefore the distribution in the heavy-top limit is computed with the respective scale of the top contribution $Q_{\text {sh}}=Q_t$. As we observed before the Monte Carlo dependence is quite small; therefore, we only consider the Pythia8 shower. For reference the black solid curve is the same as in Fig. 2 with $Q_{\text {sh}}=m_{h}/2$ for all contributions. We compare it to the scales choices proposed in Ref. [69], which imply setting the shower scale of the bottom contribution (including the interference) to the bottom mass following Eq. (2) (red dotted curve). For the blue dashed curve with points we chose the HMW scales determined in Ref. [72] which can be found in Table 1 of that paper, applying a three-scale approach according to Eq. (3) ($Q_t=49$ GeV, $Q_{tb}=34$ GeV and $Q_b=23$ GeV). The green dash-dotted curve with open boxes serves mostly for comparison with previous Herwig6 results [111] which were computed with the scales of Ref. [69] as well.

For the $p_T$ distribution in Fig. 3a, the change of the scale of the bottom contribution to $Q_b=m_b$ has a significant impact on the mass effects at small and intermediate transverse momenta. It develops an extremely steep drop at small transverse momenta which due to unitarity affects also the intermediate $p_T$-range in the opposite direction. The benefit of the usage of such a low scale is clearly disputable. While the Herwig6 curve agrees rather well with previous result of Ref. [111] becoming flat for $p_T\lesssim 5$ GeV, the Pythia8 curve develops a steep increase in this region. This signals a significant Monte Carlo dependence at very small $p_T$ which is not observed for larger $Q_b$ scales. Furthermore, the rigorously low value also poses a technical problem in the code regarding the fact that the default shower scale choice in MadGraph5_aMC@NLO, as explained in Sect. 3.3, is a distribution. In order to solve this problem, we had to use a fixed value of $Q_{\text {sh}}=Q_b$ by setting $\mathtt{frac\_low}=\mathtt{frac\_upp}=Q_b/m_{h}$ and $\mathtt{scaleMClow}=\mathtt{scaleMCdelta}=0$.

Considering the HMW scales, the scale of the bottom contribution is not chosen at such low values. We find that the mass effects in this case (blue dashed line with points) are rather similar to the ones where all scales are set to $m_{h}/2$ (black solid line), although the individual HMW scales being quite different from this value. Looking at the rapidity distribution in Fig. 3b, on the other hand, we observe the expected feature of being essentially insensitive to any choice of the respective shower scales. We shall note at this point that simply due to their inclusion in the default analysis we were able to produce a large number of further observables at no additional computing cost.

**Fig. 4:**
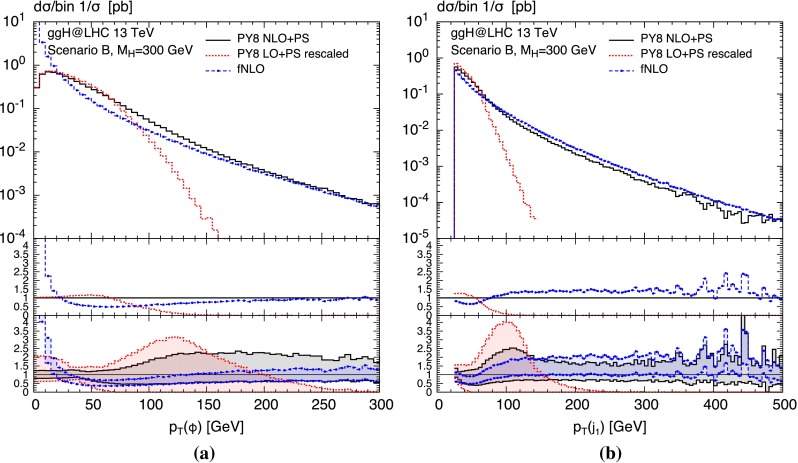
Transverse momentum distribution of **a** the heavy Higgs boson and **b** the associated hardest jet computed in a *bottom* dominated scenario of the 2HDM (see text for details). The graphical notation is the following: the *black solid curve* shows Pythia8 at NLO $+$ PS, the *red dotted curve* is the same at LO $+$ PS (normalized the NLO) and the *blue dashed one with points* corresponds to the fixed-order curve at NLO

**Fig. 5:**
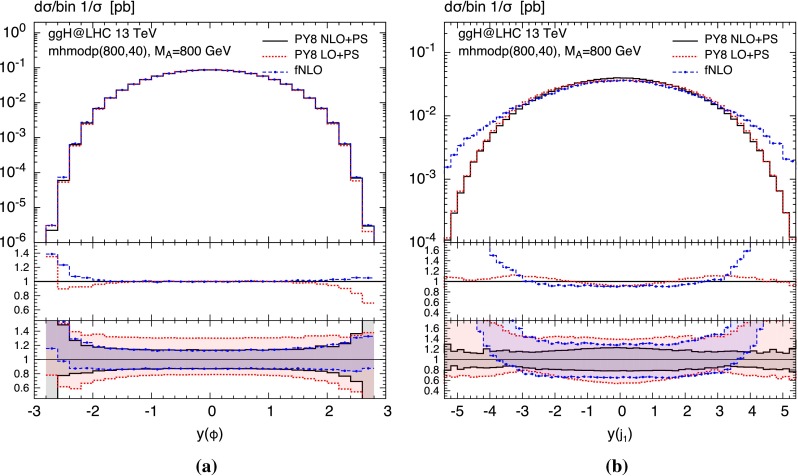
Rapidity distribution of **a** the pseudo-scalar Higgs boson and **b** the associated hardest jet computed in the $m_h^{\text {mod}+}$ scenario [114] of the MSSM with $M_A=800$ GeV und $\tan \beta =40$. The graphical notation is the same as in Fig. 4. All *curves* are normalized so that their bins add up to 1

To demonstrate the range of applicability of aMCSusHi, we consider two realistic BSM scenarios in Figs. 4 and 5: the heavy Higgs boson in Scenario B of Ref. [113] (a bottom dominated 2HDM scenario); and the pseudo-scalar Higgs boson in the $m_h^{\text {mod}+}$ (800, 40) MSSM scenario [114] defined in Table 2 of Ref. [72]. The corresponding input files can be found in the folder $\langle$ggH-folder$\rangle$/Cards.

In Fig. 4 we study the transverse momentum distributions of the Higgs and the hardest jet, while Fig. 5 depicts their rapidity distributions. In both cases we apply the HMW scales of Ref. [72]. At low transverse momenta the $p_T$ distributions have similar shapes comparing the red dotted (LO $+$ PS) to the black solid curves (NLO $+$ PS). This can easily be inferred from the first inset where all curves are normalized to the black solid line in the main frame. However, it is well known that at the LO $+$ PS $p_T$ distributions yield unphysical results for transverse momenta beyond the shower scales indicated by a steep drop. Note that both curves are normalized to the same (the NLO) cross section. Continuing the comparison at hand, we observe a significant reduction of the scale uncertainties shown in the lower inset, where the bands are obtained by dividing the upper and lower bound of the respective cross section by the same central cross section as in the first inset. The uncertainties correspond to the independent variation of all unphysical scales ($\mu _\mathrm{R}$, $\mu _\mathrm{F}$, $Q_t$, $Q_b$, $Q_{tb}$) by a factor of two. Comparing NLO $+$ PS to the NLO fixed-order result denoted by fNLO, we observe the expected matching toward large transverse momenta.

In order to compare shapes, the rapidity distributions in Fig. 5 are normalized in a way that the sum of their bins yields 1. We see that for the Higgs rapidity in Fig. 5a all curves agree extremely well in terms of shape up to the forward region in which, nevertheless, the deviations are still well within the respective uncertainty bands.^12^ For the rapidity distribution of the hardest jet the same is true for the two showered results, while the fNLO distribution, on the other hand, agrees only in the central region $|y(j_1)|\lesssim 3$, but it features a significant enhancement when the hardest jet is more forward. In this region the cross section will receive large effects of collinear radiation which renders the shower to yield the more reliable description.

## Conclusions

In this article we presented the new tool aMCSusHi which is a link between MadGraph5_aMC@NLO and SusHi for the computation of Higgs cross sections in gluon fusion at hadron colliders. The code gives NLO $+$ PS accurate results in the SM, 2HDM and MSSM. The inputs in the MSSM are conveniently controlled through a link to FeynHiggs. We discussed the specific treatment of the shower scale in MadGraph5_aMC@NLO and pointed out its special role in the context of gluon-induced Higgs production. In the phenomenological part we study the impact of different shower scale choices on the mass effects in the SM. Furthermore, we studied results for 2HDM and MSSM Higgs production as an application of aMCSusHi. The aMCSusHi script can be downloaded from https://cp3.irmp.ucl.ac.be/projects/madgraph/wiki/aMCSushi.

As an outlook, one may improve the SM prediction for the Higgs production mode through gluon fusion by merging the NLO $+$ PS cross section for $gg\rightarrow h$ in the full theory with higher multiplicities computed in the heavy-top effective field theory. This is certainly beyond the scope of the present paper and is left for a future publication.

## Footnotes

1 See Refs. [3–5] for a theoretical overview.

2 Note that in theories with an enhanced bottom Yukawa the associated Higgs production with bottom quarks becomes relevant; see Refs. [9–17] and references therein.

3 aMCSusHi can be downloaded under https://cp3.irmp.ucl.ac.be/projects/madgraph/wiki/aMCSushi.

4 SusHi has recently been extended to include NMSSM Higgs production [75], which may be made available in aMCSusHi in the future.

5 See the README file how this can easily be obtained with the MadGraph5_aMC@NLO script.

6 Please note that even in cases where the compilation of FeynHiggs or SusHi fails, the linking may still work fine as long as the files libFH.a and libsushi.a have been created in the corresponding library folders.

7 Note that only in the on-shell scheme the automatic MadGraph5_aMC@NLO reweighting for $\mu _\mathrm{R}$ is functionable.

8 Bear in mind that this has to be taken into account to identify the Higgs in the analysis of the showered events.

9 A link to 2HDMC [99] with the corresponding input convention is currently not supported.

10 See Refs. [100–105] for further information.

11 For further information we refer the reader to Sect. 2.4.4 of Ref. [73].

12 Note that the Higgs reaches its kinematical limit slightly before $|y(\phi )|\sim 3$ already.

## References

[1] ATLAS Collaboration, G. Aad et al., Observation of a new particle in the search for the Standard Model Higgs boson with the ATLAS detector at the LHC. Phys. Lett. B **716**, 1–29 (2012). arXiv:1207.7214

[2] CMS Collaboration, S. Chatrchyan et al., Observation of a new boson at a mass of 125 GeV with the CMS experiment at the LHC. Phys. Lett. B **716**, 30–61 (2012). arXiv:1207.7235

[3] LHC Higgs Cross Section Working Group Collaboration, S. Dittmaier et al., Handbook of LHC Higgs cross sections: 1. Inclusive observables. arXiv:1101.0593

[4] LHC Higgs Cross Section Working Group Collaboration, S. Dittmaier et al., Handbook of LHC Higgs cross sections: 2. Differential distributions. arXiv:1201.3084

[5] LHC Higgs Cross Section Working Group Collaboration, S. Heinemeyer et al., Handbook of LHC Higgs cross sections: 3. Higgs properties. arXiv:1307.1347

[6] Georgi H, Glashow S, Machacek M, Nanopoulos DV (1978). Higgs bosons from two gluon annihilation in proton–proton collisions. Phys. Rev. Lett..

[7] Dawson S (1991). Radiative corrections to Higgs boson production. Nucl. Phys. B.

[8] Djouadi A, Spira M, Zerwas P (1991). Production of Higgs bosons in proton colliders: QCD corrections. Phys. Lett. B.

[9] J.M. Campbell, R.K. Ellis, F. Maltoni, S. Willenbrock, Higgs-Boson production in association with a single bottom quark. Phys. Rev. D **67**, 095002 (2003). arXiv:hep-ph/0204093

[10] R.V. Harlander, W.B. Kilgore, Higgs boson production in bottom quark fusion at next-to-next-to leading order. Phys. Rev. D **68**, 013001 (2003). arXiv:hep-ph/0304035

[11] S. Dittmaier, M. Krämer, M. Spira, Higgs radiation off bottom quarks at the Tevatron and the CERN LHC. Phys. Rev. D **70**, 074010 (2004). arXiv:hep-ph/0309204 10.1103/PhysRevLett.87.201805 11690466

[12] S. Dawson, C. Jackson, L. Reina, D. Wackeroth, Exclusive Higgs boson production with bottom quarks at hadron colliders. Phys. Rev. D **69**, 074027 (2004). arXiv:hep-ph/0311067 10.1103/PhysRevLett.94.031802 15698250

[13] Harlander RV, Ozeren KJ, Wiesemann M (2010). Higgs plus jet production in bottom quark annihilation at next-to-leading order. Phys. Lett. B.

[14] Harlander R, Wiesemann M (2012). Jet-veto in bottom-quark induced Higgs production at next-to-next-to-leading order. JHEP.

[15] Bühler S, Herzog F, Lazopoulos A, Müller R (2012). The fully differential hadronic production of a Higgs boson via bottom quark fusion at NNLO. JHEP.

[16] R.V. Harlander, A. Tripathi, M. Wiesemann, Higgs production in bottom quark annihilation: transverse momentum distribution at NNLO $$+$$ NNLL. Phys. Rev. D **90**(1), 015017 (2014). arXiv:1403.7196

[17] Wiesemann M, Frederix R, Frixione S, Hirschi V, Maltoni F, Torrielli P (2015). Higgs production in association with bottom quarks. JHEP.

[18] M. Spira, A. Djouadi, D. Graudenz, P. Zerwas, Higgs boson production at the LHC. Nucl. Phys. B **453**, 17–82 (1995). arXiv:hep-ph/9504378

[19] R. Harlander, P. Kant, Higgs production and decay: analytic results at next-to-leading order QCD. JHEP **0512**, 015 (2005). arXiv:hep-ph/0509189

[20] R.V. Harlander, M. Steinhauser, Hadronic Higgs production and decay in supersymmetry at next-to-leading order. Phys. Lett. B **574**, 258–268 (2003). arXiv:hep-ph/0307346

[21] R.V. Harlander, M. Steinhauser, Supersymmetric Higgs production in gluon fusion at next-to-leading order. JHEP **0409**, 066 (2004). arXiv:hep-ph/0409010

[22] R. Harlander, M. Steinhauser, Effects of SUSY QCD in hadronic Higgs production at next-to-next-to-leading order. Phys. Rev. D **68**, 111701 (2003). arXiv:hep-ph/0308210

[23] R.V. Harlander, F. Hofmann, Pseudo-scalar Higgs production at next-to-leading order SUSY-QCD. JHEP **0603**, 050 (2006). arXiv:hep-ph/0507041

[24] M. Mühlleitner, M. Spira, Higgs boson production via gluon fusion: squark loops at NLO QCD. Nucl. Phys. B **790**, 1–27 (2008). arXiv:hep-ph/0612254

[25] U. Aglietti, R. Bonciani, G. Degrassi, A. Vicini, Analytic results for virtual QCD corrections to Higgs production and decay. JHEP **0701**, 021 (2007). arXiv:hep-ph/0611266

[26] Bonciani R, Degrassi G, Vicini A (2007). Scalar particle contribution to Higgs production via gluon fusion at NLO. JHEP.

[27] Degrassi G, Slavich P (2008). On the NLO QCD corrections to Higgs production and decay in the MSSM. Nucl. Phys. B.

[28] Anastasiou C, Beerli S, Daleo A (2008). The two-loop QCD amplitude $$gg \rightarrow h, H$$ in the Minimal Supersymmetric Standard Model. Phys. Rev. Lett..

[29] Harlander RV, Hofmann F, Mantler H (2011). Supersymmetric Higgs production in gluon fusion. JHEP.

[30] Degrassi G, Slavich P (2010). NLO QCD bottom corrections to Higgs boson production in the MSSM. JHEP.

[31] M. Mühlleitner, H. Rzehak, M. Spira, SUSY-QCD corrections to MSSM Higgs boson production via gluon fusion. PoS RADCOR2009 043 (2010). arXiv:1001.3214

[32] Degrassi G, Di Vita S, Slavich P (2011). NLO QCD corrections to pseudoscalar Higgs production in the MSSM. JHEP.

[33] Bagnaschi E, Degrassi G, Slavich P, Vicini A (2012). Higgs production via gluon fusion in the POWHEG approach in the SM and in the MSSM. JHEP.

[34] Degrassi G, Di Vita S, Slavich P (2012). On the NLO QCD corrections to the production of the heaviest neutral Higgs scalar in the MSSM. Eur. Phys. J. C.

[35] R.V. Harlander, W.B. Kilgore, Next-to-next-to-leading order Higgs production at hadron colliders. Phys. Rev. Lett. **88**, 201801 (2002). arXiv:hep-ph/0201206 10.1103/PhysRevLett.88.201801 12005555

[36] C. Anastasiou, K. Melnikov, Higgs boson production at hadron colliders in NNLO QCD. Nucl. Phys. B **646**, 220–256 (2002). arXiv:hep-ph/0207004

[37] V. Ravindran, J. Smith, W.L. van Neerven, NNLO corrections to the total cross-section for Higgs boson production in hadron–hadron collisions. Nucl. Phys. B **665**, 325–366 (2003). arXiv:hep-ph/0302135

[38] C. Anastasiou, K. Melnikov, F. Petriello, Higgs boson production at hadron colliders: differential cross sections through next-to-next-to-leading order. Phys. Rev. Lett. **93**, 262002 (2004). arXiv:hep-ph/0409088 10.1103/PhysRevLett.93.262002 15697969

[39] S. Catani, M. Grazzini, An NNLO subtraction formalism in hadron collisions and its application to Higgs boson production at the LHC. Phys. Rev. Lett. **98**, 222002 (2007). arXiv:hep-ph/0703012 10.1103/PhysRevLett.98.222002 17677837

[40] Grazzini M (2008). NNLO predictions for the Higgs boson signal in the $$H\rightarrow WW \rightarrow l\nu l\nu $$ and $$H \rightarrow ZZ \rightarrow 4l$$ decay channels. JHEP.

[41] S. Catani, D. de Florian, M. Grazzini, P. Nason, Soft gluon resummation for Higgs boson production at hadron colliders. JHEP **0307**, 028 (2003). arXiv:hep-ph/0306211

[42] A. Idilbi, X.-D. Ji, J.-P. Ma, F. Yuan, Threshold resummation for Higgs production in effective field theory. Phys. Rev. D **73**, 077501 (2006). arXiv:hep-ph/0509294

[43] V. Ravindran, Higher-order threshold effects to inclusive processes in QCD. Nucl. Phys. B **752**, 173–196 (2006). arXiv:hep-ph/0603041

[44] Ahrens V, Becher T, Neubert M, Yang LL (2009). Renormalization-group improved prediction for Higgs production at hadron colliders. Eur. Phys. J. C.

[45] A. Djouadi, P. Gambino, Leading electroweak correction to Higgs boson production at proton colliders. Phys. Rev. Lett. **73**, 2528–2531 (1994). arXiv:hep-ph/9406432 10.1103/PhysRevLett.73.2528 10057083

[46] G. Degrassi, F. Maltoni, Two-loop electroweak corrections to Higgs production at hadron colliders. Phys. Lett. B **600**, 255–260 (2004). arXiv:hep-ph/0407249

[47] Anastasiou C, Boughezal R, Petriello F (2009). Mixed QCD-electroweak corrections to Higgs boson production in gluon fusion. JHEP.

[48] U. Aglietti, R. Bonciani, G. Degrassi, A. Vicini, Two loop light fermion contribution to Higgs production and decays. Phys. Lett. B **595**, 432–441 (2004). arXiv:hep-ph/0404071

[49] Actis S, Passarino G, Sturm C, Uccirati S (2008). NLO electroweak corrections to Higgs boson production at hadron colliders. Phys. Lett. B.

[50] Bonciani R, Degrassi G, Vicini A (2011). On the generalized harmonic polylogarithms of one complex variable. Comput. Phys. Commun..

[51] Anastasiou C, Duhr C, Dulat F, Furlan E, Gehrmann T, Herzog F, Mistlberger B (2014). Higgs boson gluonfusion production at threshold in N$$^3$$LO QCD. Phys. Lett. B.

[52] C. Anastasiou, C. Duhr, F. Dulat, E. Furlan, T. Gehrmann, F. Herzog, B. Mistlberger, Higgs boson gluon-fusion production beyond threshold in N3LO QCD. arXiv:1411.3584 10.1103/PhysRevLett.114.212001 26066428

[53] C. Anastasiou, C. Duhr, F. Dulat, F. Herzog, B. Mistlberger, Higgs boson gluon-fusion production in N3LO QCD. arXiv:1503.06056 10.1103/PhysRevLett.114.212001 26066428

[54] Marzani S, Ball RD, Del Duca V, Forte S, Vicini A (2008). Higgs production via gluon–gluon fusion with finite top mass beyond next-to-leading order. Nucl. Phys. B.

[55] Harlander RV, Ozeren KJ (2009). Top mass effects in Higgs production at next-to-next-to-leading order QCD: virtual corrections. Phys. Lett. B.

[56] Harlander RV, Ozeren KJ (2009). Finite top mass effects for hadronic Higgs production at next-to-next-to-leading order. JHEP.

[57] Harlander RV, Mantler H, Marzani S, Ozeren KJ (2010). Higgs production in gluon fusion at next-to-next-to-leading order QCD for finite top mass. Eur. Phys. J. C.

[58] Pak A, Rogal M, Steinhauser M (2010). Finite top quark mass effects in NNLO Higgs boson production at LHC. JHEP.

[59] Pak A, Rogal M, Steinhauser M (2011). Production of scalar and pseudo-scalar Higgs bosons to next-to-next-to-leading order at hadron colliders. JHEP.

[60] Harlander RV, Neumann T, Ozeren KJ, Wiesemann M (2012). Top-mass effects in differential Higgs production through gluon fusion at order $$\alpha _s^4$$. JHEP.

[61] Neumann T, Wiesemann M (2014). Finite top-mass effects in gluon-induced Higgs production with a jet-veto at NNLO. JHEP.

[62] P. Nason, A New method for combining NLO QCD with shower Monte Carlo algorithms. JHEP **0411**, 040 (2004). arXiv:hep-ph/0409146

[63] H. Mantler, M. Wiesemann, Top- and bottom-mass effects in hadronic Higgs production at small transverse momenta through LO $$+$$ NLL. Eur. Phys. J. C **73**(6), 2467 (2013). arXiv:1210.8263

[64] S. Frixione, B.R. Webber, Matching NLO QCD computations and parton shower simulations. JHEP **0206**, 029 (2002). arXiv:hep-ph/0204244

[65] S. Frixione, F. Stoeckli, P. Torrielli, B.R. Webber, C.D. White, MC@NLO 4.10. http://www.hep.phy.cam.ac.uk/theory/webber/MCatNLO/. Accessed 1 June 2015

[66] S. Frixione, F. Stoeckli, P. Torrielli, B.R. Webber, C.D. White, The MC@NLO 4.0 event generator. arXiv:1010.0819

[67] Frixione S, Stoeckli F, Torrielli P, Webber BR (2011). NLO QCD corrections in Herwig++ with MC@NLO. JHEP.

[68] Banfi A, Monni PF, Zanderighi G (2014). Quark masses in Higgs production with a jet veto. JHEP.

[69] Grazzini M, Sargsyan H (2013). Heavy-quark mass effects in Higgs boson production at the LHC. JHEP.

[70] Hamilton K, Nason P, Re E, Zanderighi G (2013). NNLOPS simulation of Higgs boson production. JHEP.

[71] K. Hamilton, P. Nason, G. Zanderighi, Finite quark-mass effects in the NNLOPS POWHEG $$+$$ MiNLO Higgs generator. arXiv:1501.04637

[72] Harlander RV, Mantler H, Wiesemann M (2014). Transverse momentum resummation for Higgs production via gluon fusion in the MSSM. JHEP.

[73] Alwall J, Frederix R, Frixione S, Hirschi V, Maltoni F, Mattelaer O, Shao H-S, Stelzer T, Torrielli P, Zaro M (2014). The automated computation of tree-level and next-to-leading order differential cross sections, and their matching to parton shower simulations. JHEP.

[74] Harlander RV, Liebler S, Mantler H (2013). SusHi: a program for the calculation of Higgs production in gluon fusion and bottom-quark annihilation in the Standard Model and the MSSM. Comput. Phys. Commun..

[75] S. Liebler, Neutral Higgs production at proton colliders in the CP-conserving NMSSM. arXiv:1502.07972

[76] G. Corcella, I. Knowles, G. Marchesini, S. Moretti, K. Odagiri, P. Richardson, M.H. Seymour, B.R. Webber, HERWIG 6: an event generator for hadron emission reactions with interfering gluons (including supersymmetric processes). JHEP **0101**, 010 (2001). arXiv:hep-ph/0011363

[77] G. Corcella, I. Knowles, G. Marchesini, S. Moretti, K. Odagiri, P. Richardson, M.H. Seymour, B.R. Webber, HERWIG 6.5 release note. arXiv:hep-ph/0210213

[78] T. Sjostrand, S. Mrenna, P.Z. Skands, PYTHIA 6.4 physics and manual. JHEP **0605**, 026 (2006). arXiv:hep-ph/0603175

[79] Sjostrand T, Mrenna S, Skands PZ, Brief A (2008). Introduction to PYTHIA 8.1. Comput. Phys. Commun..

[80] Bahr M, Gieseke S, Gigg M, Grellscheid D, Hamilton K, Latunde-Dada O, Platzer S, Richardson P (2008). Herwig++ physics and manual. Eur. Phys. J. C.

[81] J. Bellm, S. Gieseke, D. Grellscheid, A. Papaefstathiou, S. Platzer, P. Richardson, C. Rohr, T. Schuh, Herwig++ 2.7 release note. arXiv:1310.6877

[82] Degrande C, Duhr C, Fuks B, Grellscheid D, Mattelaer O, Reiter T (2012). UFO - the Universal FeynRules Output. Comput. Phys. Commun..

[83] Christensen ND, Duhr C (2009). FeynRules - Feynman rules made easy. Comput. Phys. Commun..

[84] Christensen ND, de Aquino P, Degrande C, Duhr C, Fuks B, Herquet M, Maltoni F, Schumann S (2011). A comprehensive approach to new physics simulations. Eur. Phys. J. C.

[85] Alloul A, Christensen ND, Degrande C, Duhr C, Fuks B (2014). FeynRules 2.0 - a complete toolbox for tree-level phenomenology. Comput. Phys. Commun..

[86] S. Heinemeyer, W. Hollik, G. Weiglein, FeynHiggs: a program for the calculation of the masses of the neutral CP even Higgs bosons in the MSSM. Comput. Phys. Commun. **124**, 76–89 (2000). arXiv:hep-ph/9812320

[87] S. Heinemeyer, W. Hollik, G. Weiglein, The masses of the neutral CP - even Higgs bosons in the MSSM: accurate analysis at the two loop level. Eur. Phys. J. C **9**, 343–366 (1999). arXiv:hep-ph/9812472

[88] G. Degrassi, S. Heinemeyer, W. Hollik, P. Slavich, G. Weiglein, Towards high precision predictions for the MSSM Higgs sector. Eur. Phys. J. C **28**, 133–143 (2003). arXiv:hep-ph/0212020

[89] M. Frank, T. Hahn, S. Heinemeyer, W. Hollik, H. Rzehak, G. Weiglein, The Higgs boson masses and mixings of the complex MSSM in the Feynman-diagrammatic approach. JHEP **0702**, 047 (2007). arXiv:hep-ph/0611326

[90] T. Hahn, S. Heinemeyer, W. Hollik, H. Rzehak, G. Weiglein, High-precision predictions for the light CP-even Higgs boson mass of the Minimal Supersymmetric Standard Model. Phys. Rev. Lett. **112**(14), 141801 (2014). arXiv:1312.4937 10.1103/PhysRevLett.112.141801 24765944

[91] G. Degrassi, P. Slavich, F. Zwirner, On the neutral Higgs boson masses in the MSSM for arbitrary stop mixing. Nucl. Phys. B **611**, 403–422 (2001). arXiv:hep-ph/0105096

[92] A. Brignole, G. Degrassi, P. Slavich, F. Zwirner, On the $${\cal {O}} (\alpha _t^2)$$ two loop corrections to the neutral Higgs boson masses in the MSSM. Nucl. Phys. B **631**, 195–218 (2002). arXiv:hep-ph/0112177

[93] A. Brignole, G. Degrassi, P. Slavich, F. Zwirner, On the two loops bottom corrections to the neutral Higgs boson masses in the MSSM. Nucl. Phys. B **643**, 79–92 (2002). arXiv:hep-ph/0206101

[94] A. Dedes, G. Degrassi, P. Slavich, On the two loop Yukawa corrections to the MSSM Higgs boson masses at large tan beta. Nucl. Phys. B **672**, 144–162 (2003). arXiv:hep-ph/0305127

[95] S. Heinemeyer, W. Hollik, H. Rzehak, G. Weiglein, High-precision predictions for the MSSM Higgs sector at $${\cal {O}} (\alpha _b \alpha _s)$$. Eur. Phys. J. C **39**, 465–481 (2005). arXiv:hep-ph/0411114

[96] Heinemeyer S, Hollik W, Rzehak H, Weiglein G (2007). The Higgs sector of the complex MSSM at two-loop order: QCD contributions. Phys. Lett. B.

[97] P.Z. Skands et al., SUSY Les Houches accord: interfacing SUSY spectrum calculators, decay packages, and event generators. JHEP **0407**, 036 (2004). arXiv:hep-ph/0311123

[98] Bagnaschi E, Harlander R, Liebler S, Mantler H, Slavich P, Vicini A (2014). Towards precise predictions for Higgs-boson production in the MSSM. JHEP.

[99] Eriksson D, Rathsman J, Stal O (2010). 2HDMC: two-Higgs-doublet model calculator physics and manual. Comput. Phys. Commun..

[100] M. Carena, D. Garcia, U. Nierste, C.E. Wagner, Effective Lagrangian for the $$\bar{t} b H^{+}$$ interaction in the MSSM and charged Higgs phenomenology. Nucl. Phys. B **577**, 88–120 (2000). arXiv:hep-ph/9912516

[101] J. Guasch, P. Hafliger, M. Spira, MSSM Higgs decays to bottom quark pairs revisited. Phys. Rev. D **68**, 115001 (2003). arXiv:hep-ph/0305101

[102] Noth D, Spira M (2011). Supersymmetric Higgs Yukawa couplings to bottom quarks at next-to-next-to-leading order. JHEP.

[103] Noth D, Spira M (2008). Higgs boson couplings to bottom quarks: two-loop supersymmetry-QCD corrections. Phys. Rev. Lett..

[104] Hofer L, Nierste U, Scherer D (2009). Resummation of tan-beta-enhanced supersymmetric loop corrections beyond the decoupling limit. JHEP.

[105] Mihaila L, Reisser C (2010). $${\cal {O}} (\alpha _s^2)$$ corrections to fermionic Higgs decays in the MSSM. JHEP.

[106] R.V. Harlander, S. Liebler, H. Mantler, SusHi 1.5.0: a program for the calculation of Higgs production in gluon fusion and bottom-quark annihilation in the Standard Model, the 2HDM, the MSSM and the NMSSM. http://sushi.hepforge.org/manual.html. Accessed 1 June 2015

[107] FeynHiggs man pages. http://wwwth.mpp.mpg.de/members/heinemey/feynhiggs/cFeynHiggs.html. Accessed 1 June 2015

[108] E. Bagnaschi, A. Vicini, The Higgs transverse momentum distribution in gluon fusion as a multiscale problem (2015). arXiv:1505.00735 [hep-ph]

[109] E. Bagnaschi, R. V. Harlander, H. Mantler, A. Vicini, M. Wiesemann, Resummation effects in Higgs transverse momentum distribution in the SM and beyond. (to be published elsewhere)

[110] Frederix R, Frixione S, Hirschi V, Maltoni F, Pittau R, Torrielli P (2012). Four-lepton production at hadron colliders: aMC@NLO predictions with theoretical uncertainties. JHEP.

[111] S. Frixione, Talk given at the ggF meeting on Higgs pT, CERN (2013). http://indico.cern.ch/event/263472/contribution/3/material/slides/. Accessed 1 June 2015

[112] Martin A, Stirling W, Thorne R, Watt G (2009). Parton distributions for the LHC. Eur. Phys. J. C.

[113] R. Harlander, M. Mühlleitner, J. Rathsman, M. Spira, O. Stål, Interim recommendations for the evaluation of Higgs production cross sections and branching ratios at the LHC in the two-Higgs-doublet model. arXiv:1312.5571

[114] M. Carena, S. Heinemeyer, O. Stål, C. Wagner, G. Weiglein, MSSM Higgs boson searches at the LHC: benchmark scenarios after the discovery of a Higgs-like particle. Eur. Phys. J. C **73**(9), 2552 (2013). arXiv:1302.7033

